# Hepatocellular carcinoma with renal invasion and its multimodal management: a rare case report

**DOI:** 10.3389/fonc.2026.1764947

**Published:** 2026-03-24

**Authors:** Weiguo Liu, Moli Chen, Xue Wei, Peng Wang

**Affiliations:** Fourth Department of Comprehensive Oncology, Hefei Cancer Hospital, Chinese Academy of Sciences, Hefei, Anhui, China

**Keywords:** case report, hepatocellular carcinoma, multimodal therapy, rare tumor invasion, renal invasion

## Abstract

This case report presents a 60-year-old female diagnosed with hepatocellular carcinoma (HCC) and direct renal invasion. Imaging identified a large hepatic mass involving the right kidney, which was confirmed histopathologically and supported by elevated alpha-fetoprotein (AFP) levels. Treatment involved a multimodal strategy combining transarterial chemoembolization (TACE), fractionated radiotherapy, and combination immunotherapy with camrelizumab plus apatinib. Follow-up imaging and AFP trends showed marked tumor regression and biochemical improvement. This case highlights the rare entity of direct renal invasion from HCC and provides practical insights into its diagnosis and comprehensive management. It illustrates the clinical feasibility and potential benefit of a combined locoregional and systemic strategy in advanced HCC with such uncommon extrahepatic renal involvement, underscoring the importance of individualized treatment in these complex presentations.

## Introduction

Hepatocellular carcinoma (HCC) is the fourth most common cancer and the second leading cause of cancer-related deaths in China, posing a significant challenge to public health (1). The carcinogenic factors of HCC are diverse, including hepatitis B virus infection, alcohol consumption, and metabolic diseases. As HCC progresses, distant metastasis can occur, commonly to sites such as the lungs, abdominal lymph nodes, and bones. Invasion to rare sites can also occur (2, 3). This article reports a case of HCC with renal invasion and subsequent treatment.

## Case report

A 60-year-old female patient presented to our hospital in early March 2025 due to a right renal mass discovered on abdominal CT. The patient reported intermittent mild right lumbar soreness over the past six months, which resolved spontaneously. Urination was normal, without frequency, urgency, dysuria, or hematuria. Physical examination revealed no costovertebral angle tenderness on the right side, no palpable mass below the ribs, and no abdominal tenderness, rebound tenderness, or muscle guarding. The patient denied a history of chronic diseases such as hypertension, coronary heart disease, or diabetes, and denied a history of viral hepatitis infection.

**Figure 1:**
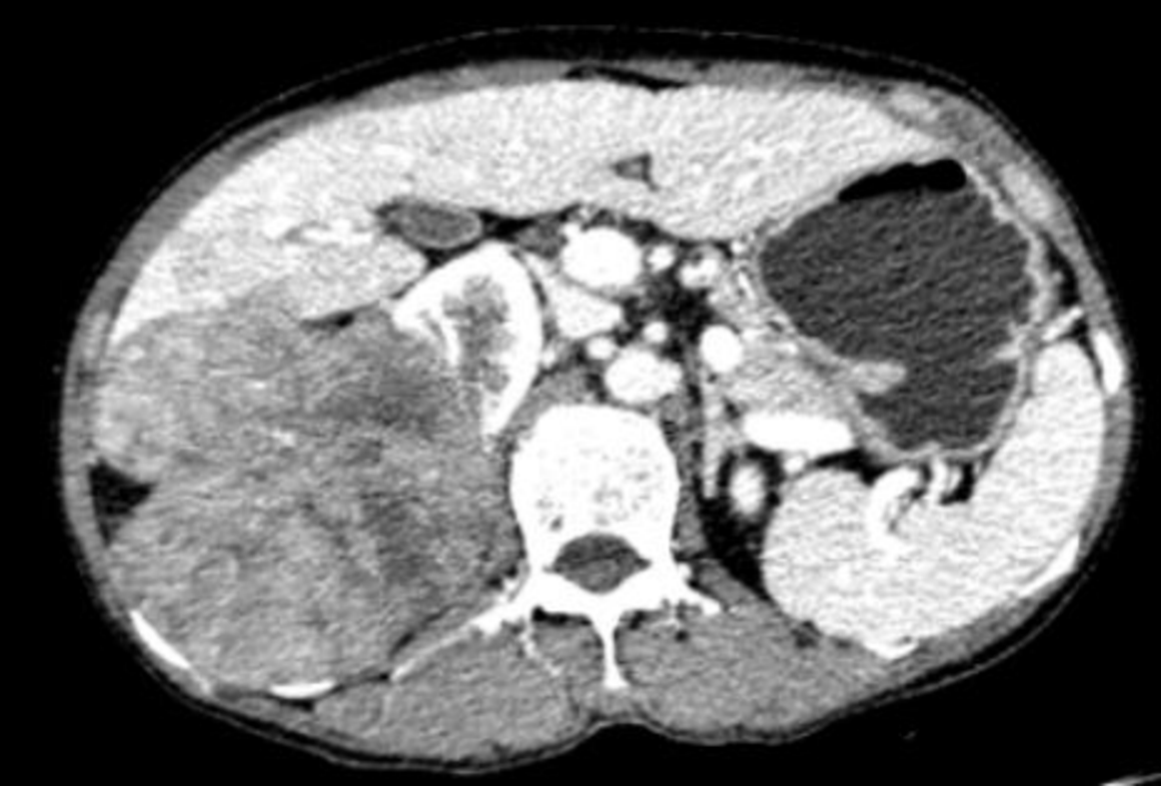
Abdominal CT, March 7, 2025: Multiple hepatic lesions, suggestive of hepatocellular carcinoma. The largest lesion measured approximately 9.3 cm × 9.1 cm at its larger cross-section, demonstrating protrusion beyond the liver to invade the right kidney.

Abdominal CT scan from our institution revealed a soft tissue density mass in the right hepatic lobe with irregular morphology and heterogeneous density, bulging beyond the hepatic contour by ≥50%. The larger cross-sectional dimension measured approximately 9.3 cm × 9.1 cm. On non-contrast images, the CT attenuation was approximately 41 HU. During the arterial phase of contrast enhancement, patchy enhancement was observed within the mass. In the portal venous and delayed phases, the degree of enhancement was lower than that of the hepatic parenchyma. Intrahepatic hepatic artery dilation was noted, with branches of the hepatic artery visible within the mass. The mass extended inferiorly, invading the right renal parenchyma, with an indistinct border from the renal parenchyma and heterogeneous enhancement on contrast-enhanced images. The right kidney appeared irregular in shape. No filling defects were observed within the right renal artery or vein, and no obvious signs of vascular invasion were present. Both adrenal glands showed no thickening. The left kidney, gallbladder, spleen, and pancreas were regular in contour with homogeneous parenchymal density, and no abnormal densities or abnormal enhancement were identified (**Figure 1**). Additionally, unenhanced and contrast-enhanced computed tomography (CT) scans of the patient’s head, neck, and chest revealed no significant space-occupying lesions. The bone scan revealed no abnormal radionuclide accumulation, ruling out bone metastasis. A PET-CT scan was not performed due to the patient’s treatment preference and financial considerations.

**Figure 2:**
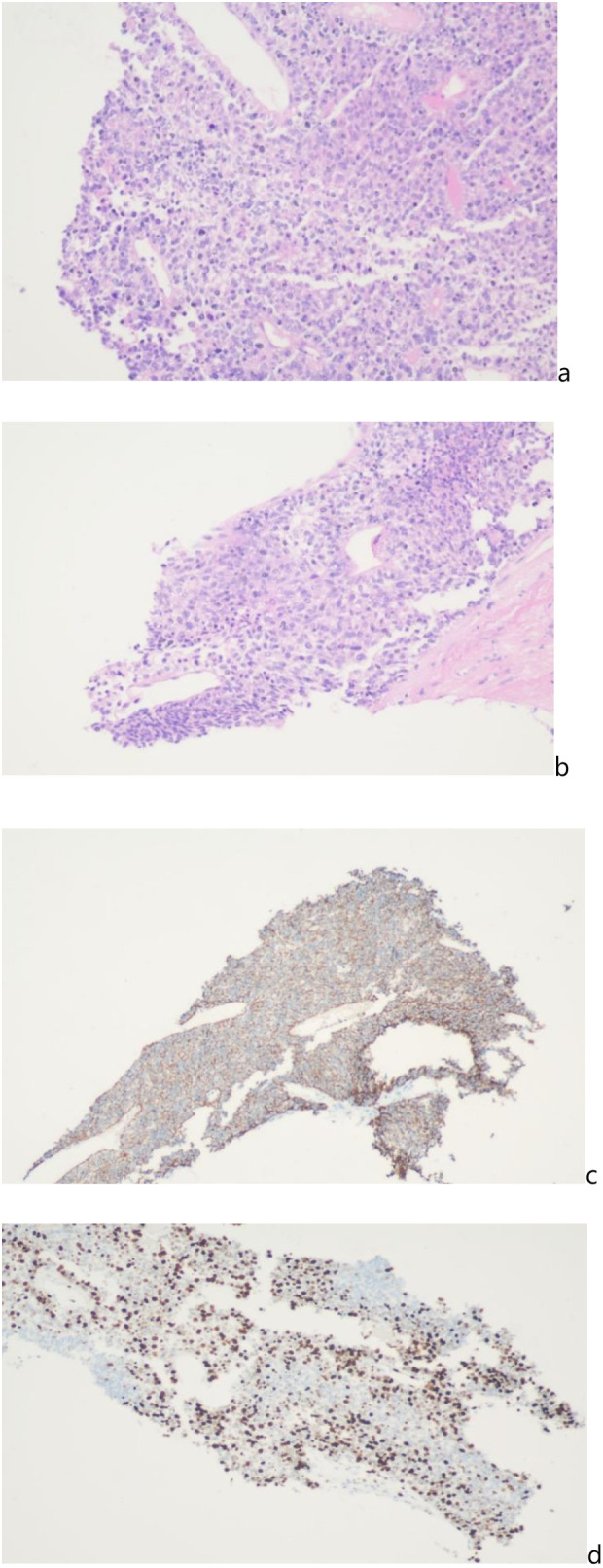
Histopathology [H&E staining, **(a, b)**] and immunohistochemistry [IHC staining, **(c, d)**] are consistent with hepatocellular carcinoma (HCC). **(a, b)** Hematoxylin and eosin (H&E) staining shows tumor cells arranged in trabecular and solid patterns with marked cellular atypia, consistent with poorly differentiated carcinoma (×100). **(c)** Immunohistochemical (IHC) staining demonstrates cytoplasmic positivity for Hepatocyte and cytoplasmic/membranous positivity for GPC-3 in tumor cells, along with diffuse CD34 positivity in tumor sinusoidal endothelial cells, supporting the diagnosis of hepatocellular carcinoma (×40). **(d)** Ki-67 IHC reveals nuclear positivity in approximately 60% of tumor cells, indicating high proliferative activity. Concurrent negativity for Vimentin, CD56, and Syn excludes tumors of mesenchymal origin or with neuroendocrine differentiation (×100).

On March 6, 2025, the serum alpha-fetoprotein (AFP) level was markedly elevated to >1,000 ng/mL. In contrast, the patient’s other tumor markers, including CEA, CA19-9, CA-125, NSE, SCC, and CA72-4, were all within normal limits (**Table 1**). This profile is highly suggestive of hepatocellular carcinoma. To confirm the pathology, an intra-abdominal mass biopsy was performed at our hospital on March 6, 2025. The pathological result indicated poorly differentiated carcinoma, which, combined with immunohistochemical findings, was consistent with hepatocellular carcinoma. The immunohistochemical profile was as follows: CK(+), Vimentin(–), Hepatocyte(+), GPC-3(+), CD34 (vascular +), CD56(–), Syn(–), and Ki-67(+, 60%) (**Figure 2**). A diagnosis of hepatocellular carcinoma with direct invasion of the right kidney was therefore established.

**Table 1:** The patient’s tumor marker profile at the initial presentation to our hospital.

Tumor marker	Result	Unit
AFP	>1000	ng/ml
NSE	12.97	ng/ml
CYFRA21-1	1.56	ng/ml
CA72-4	5.69	IU/ml
CA-125	15.63	U/ml
CA15-3	9.86	U/ml
CA19-9	11.45	U/ml
SCC	<0.20	ng/ml
HE4	68.7	pmol/ml
β-HCG	4.82	mU/ml

Based on the China Liver Cancer (CNLC) staging system, the patient was diagnosed with stage IIIb hepatocellular carcinoma. According to the Barcelona Clinic Liver Cancer (BCLC) staging system, this is classified as stage C. The CNLC guidelines recommend a multimodal treatment strategy combining systemic anti-tumor therapy, transarterial chemoembolization (TACE), and radiotherapy. Upon evaluation, given the patient’s large tumor volume, a single therapeutic modality was deemed unlikely to effectively control tumor progression. Concurrently, the patient’s liver function and general condition were adequate to tolerate a combined approach. Before treatment, the patient had a total bilirubin (TBIL) level of 8 μmol/L and an albumin (ALB) level of 35.8 g/L. In conjunction with clinical findings, the Child-Pugh grade was A. After discussing the clinical scenario and obtaining informed consent, a multimodal treatment strategy integrating pharmacotherapy, transarterial chemoembolization (TACE), and radiotherapy was selected.

On March 18, 2025, the patient underwent transcatheter arterial chemoembolization (TACE) and hepatic artery-portal vein fistula embolization (During the procedure, the interventional radiologist detected the presence of a hepatic artery-portal vein fistula on angiography and subsequently performed hepatic artery-portal vein fistula embolization) in the Interventional Department of our hospital. The procedure was successful. Postoperatively, the patient developed symptoms including fever and epigastric pain, along with elevated liver transaminases. Symptomatic management with antipyretics, analgesics, and hepatoprotective agents for transaminase reduction was administered. In accordance with the Chinese Society of Clinical Oncology (CSCO) guidelines, combined immunotherapy and targeted therapy with the regimen “camrelizumab 200mg iv every 3 weeks plus apatinib mesylate 0.5g orally once daily” was initiated on March 26, 2025. Before treatment, the patient had a total bilirubin level of 25.8 μmol/L and an albumin level of 31.5 g/L. In conjunction with clinical findings, the Child-Pugh grade was A.

The patient returned for follow-up in April 2025. An abdominal CT scan on April 8, 2025, showed the liver lesion measuring approximately 9.3 cm × 9.0 cm at its larger cross-section. The serum AFP level on the same date was 1893 ng/ml. Given the large tumor volume, radiotherapy was administered in two phases: to the superior half of the tumor volume (from the superior border of the lesion to the level of the inferior border of T12 within the lesion) in April 2025 (During radiotherapy, the patient had a total bilirubin level of 11.5 μmol/L and an albumin level of 31.3 g/L. In conjunction with clinical findings, the Child-Pugh grade was A), and to the inferior half (from the T12 inferior border level to the inferior border of the lesion) in June 2025 (During radiotherapy, the patient had a total bilirubin level of 12.8 μmol/L and an albumin level of 36 g/L. In conjunction with clinical findings, the Child-Pugh grade was A). The total dose for each phase was 3500 cGy in 10 fractions of 350 cGy per fraction. The internal target volume (ITV) encompassed the radiologically visible corresponding half of the HCC lesion, accounting for respiratory motion. The planning target volume (PTV) was defined as the ITV plus a 5-mm margin. Organs at risk (OARs) included the spinal cord, liver, and stomach, and it was required that the total dose received by OARs from both radiotherapy sessions should not exceed the cumulative dose limits. The dose constraints were as follows: spinal cord Dmax <45 Gy; liver Vmean <28 Gy and V5 <60%; stomach Dmax <45 Gy.

Treatment with the combined regimen of camrelizumab and apatinib was continued on April 23, May 19, July 1, July 28, and August 28, 2025.

**Figure 3:**
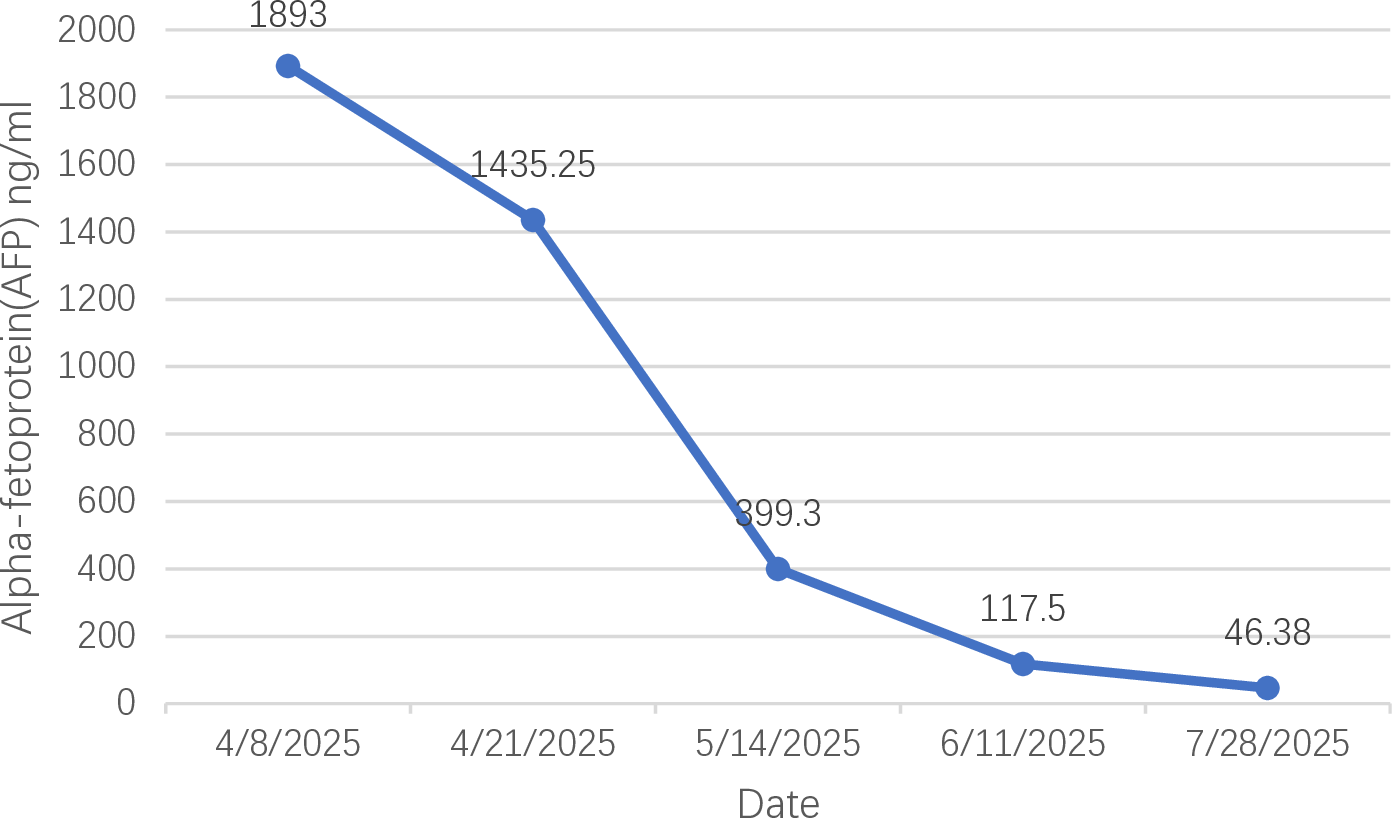
Trend of alpha-fetoprotein (AFP) levels during treatment.

**Figure 4:**
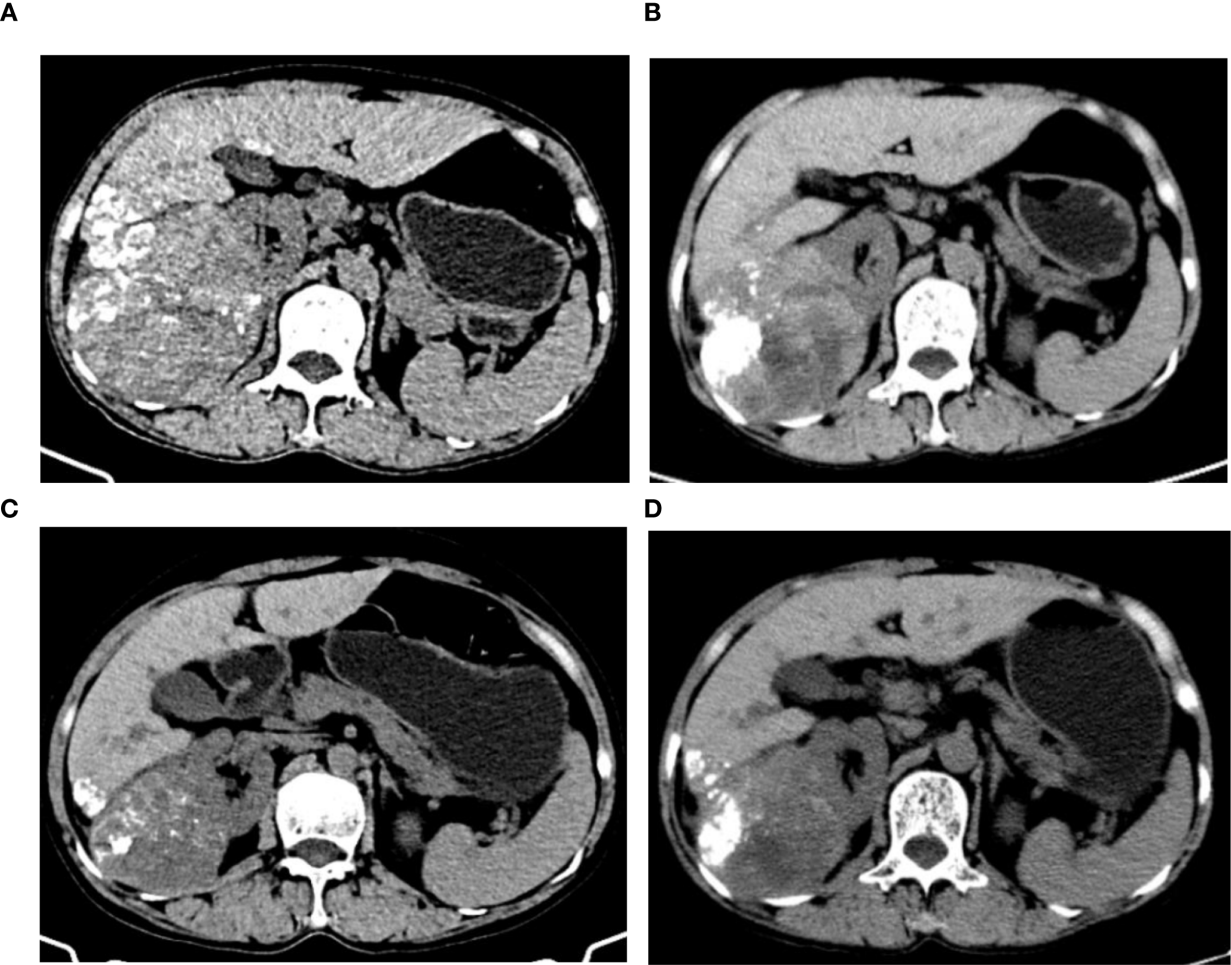
Abdominal CT scans showed a gradual reduction in the size of the liver mass [**(a)**: April 8, 2025: 9.3 cm × 9.1 cm; **(b)**: May 14, 2025: 8.2 cm × 7.3 cm; **(c)**: June 12, 2025: 8.1 cm × 6.7 cm; **(d)**: July 26, 2025: 6.2 cm × 5.9 cm].

From April to July 2025, the patient’s AFP levels continuously declined (April 8, 2025: 1893 ng/ml; April 21, 2025: 1435.25 ng/ml; May 14, 2025: 399.3 ng/ml; June 11, 2025: 117.5 ng/ml; July 28, 2025: 46.38 ng/ml) (**Figure 3**). Abdominal CT scans showed a gradual reduction in the size of the liver mass (April 8, 2025: 9.3 cm × 9.1 cm; May 14, 2025: 8.2 cm × 7.3 cm; June 12, 2025: 8.1 cm × 6.7 cm; July 26, 2025: 6.2 cm × 5.9 cm) (**Figures 4a–d**). Based on the modified Response Evaluation Criteria in Solid Tumors (mRECIST), the patient achieved a partial response. After the completion of radiotherapy, liver function was reassessed, and the patient’s Child-Pugh grade remained A, with albumin levels of 37.4, 38.1, and 37.8 g/L and total bilirubin levels of 8.9, 7.9, and 8.7 μmol/L on July 1, July 26, and August 20, 2025, respectively, and a score of 1 for the other parameters. During subsequent treatment, the patient developed hypertension (exceeding 150/95 mmHg). The patient did not report significant discomfort such as dizziness, headache, nausea, or vomiting. This hypertension was considered to be drug-induced, attributed to apatinib. Management with amlodipine besylate 5 mg orally once daily was initiated, accompanied by regular blood pressure monitoring. The patient’s blood pressure was adequately controlled, maintained at approximately 140/90 mmHg.

### Follow-up

During follow-up after August 2025, imaging revealed no significant overall change in the size of the hepatic lesion, and no evidence of extrahepatic metastasis was observed on imaging studies outside the liver (abdominal CT on September 20, 2025, showed a maximum cross-section of 6.3 cm × 6.0 cm; head, neck, and chest CT scans revealed no abnormal masses; abdominal CT on October 15, 2025, showed a maximum cross-section of 6.1 cm × 6.0 cm; abdominal CT on January 17, 2026, showed a maximum cross-section of 6.1 cm × 5.9 cm, and head, neck, and chest CT scans again showed no abnormal masses). AFP levels remained generally stable and within the normal range (September 20, 2025: 51.73 ng/mL; October 13, 2025: 56.21 ng/mL; November 30, 2025: 53.12 ng/mL; January 16, 2026: 50.63 ng/mL). Overall assessment indicated stable disease (SD).

Elevated alanine aminotransferase levels were observed, which were attributed to the combination of immunotherapy and targeted therapy; however, total bilirubin and albumin levels remained within the normal range, and the Child-Pugh grade remained A. As of the end of January 2026, the patient was in generally stable condition, with occasional transient discomfort in the right upper quadrant reported. Blood pressure was well controlled within the normal range with oral antihypertensive medication.

## Discussion

Hepatocellular carcinoma is a common malignant tumor of the liver, accounting for 75%-85% of primary liver cancers (4). Various risk factors contribute to HCC, such as alcohol, aflatoxin, and metabolic syndrome. In China, hepatitis B virus infection is the predominant risk factor, making the control of HBV infection crucial (5). Alpha-fetoprotein (AFP), a glycoprotein secreted by the yolk sac, fetal liver, and gastrointestinal tract during fetal development, is present only in trace amounts in healthy adults. Serum AFP levels are significantly elevated in HCC patients, making AFP an extremely important biomarker for the diagnosis and prognostic assessment of HCC (6, 7). Currently, studies have shown that Prothrombin induced by vitamin K absence or antagonist-II (PIVKA-II) has demonstrated significant advantages in evaluating the clinicopathological features of hepatocellular carcinoma, such as tumor size, stage, and metastasis. The combination of AFP and PIVKA-II has shown considerable promise in the clinical diagnosis and prognostic assessment of hepatocellular carcinoma (8).

Common routes of metastasis for HCC include via the portal vein, hepatic veins, and lymphatic vessels, with hematogenous spread via the hepatic veins being the most common. This may be related to the formation of hepatic vein shunts from the tumor via the hepatic venous system (9). Common sites of extrahepatic metastasis for HCC include the lungs, abdominal lymph nodes, and bones. Less common metastatic sites include the adrenal glands, diaphragm, pleura, and pancreas. Direct invasion of the kidney by HCC is extremely rare in clinical practice (2, 3).

HCC metastasis is driven by core signaling pathways that promote invasion and an immunosuppressive microenvironment. VEGF/VEGFR signaling stimulates angiogenesis and establishes a metastatic niche, while EGFR activation facilitates intrahepatic dissemination via Ras/MAPK. Concurrent dysregulation of c-Met/HGF, PDGF/PDGFR, Wnt/β-catenin, Hedgehog, and Notch pathways orchestrates epithelial-mesenchymal transition, stemness, and invasive capacity. The immunosuppressive milieu, shaped by TGF-β and etiology-specific inflammation, further enables immune evasion. Therapeutic strategies now focus on co-targeting these mechanisms, notably through combinations of anti-angiogenics (e.g. bevacizumab) with immune checkpoint inhibitors, or kinase inhibitors (e.g. cabozantinib) with immunotherapy, to simultaneously disrupt metastatic signaling and restore antitumor immunity, representing a key direction for advanced HCC management (10, 11).

Currently, several staging systems for HCC exist, based on factors such as tumor size, number, lymph node metastasis, distant metastasis, and clinical scores. These include the American Joint Committee on Cancer (AJCC) staging and the Barcelona Clinic Liver Cancer (BCLC) staging. The China Liver Cancer (CNLC) staging system incorporates performance status, liver function, extrahepatic metastasis, vascular tumor thrombus, tumor number, and tumor size, categorizing patients into stages Ia, Ib, IIa, IIb, IIIa, IIIb, and IV (4, 12). According to the CNLC staging, this patient was diagnosed with HCC stage IIIb (PS 0; Child-Pugh A; with extrahepatic metastasis). The China Liver Cancer (CNLC) staging system recommends an integrated treatment approach combining systemic anti-tumor therapy, transarterial chemoembolization (TACE), and radiotherapy.

For unresectable HCC lesions, TACE is a crucial local interventional treatment option. Currently, conventional TACE (cTACE) and drug-eluting bead TACE (DEB-TACE) are equally recommended due to similar overall survival (OS) outcomes (13).

Transarterial Radioembolization (TARE) is an interventional radiology technique used for the treatment of liver malignancies. It involves the transcatheter injection of radioactive microspheres (e.g., yttrium-90) into the tumor-supplying arteries to achieve localized tumor destruction. However, this patient was not considered for TARE because the indication for TARE is typically reserved for unresectable or non-ablatable solitary hepatocellular carcinoma (HCC) lesions ≤ 8 cm, whereas the patient’s tumor diameter exceeded 9 cm. Moreover, both the CSCO guidelines and the CNLC staging system continue to recommend TACE as the preferred modality for interventional treatment of HCC, with TARE positioned as an alternative to TACE. Given these considerations, TACE was maintained as the primary interventional approach (14).

Systemic antitumor therapy serves as a cornerstone in the management of unresectable hepatocellular carcinoma (HCC) and is increasingly trending toward combination strategies integrating targeted agents with immunotherapy. Current Chinese guidelines recommend several first-line regimens, including camrelizumab in combination with apatinib, atezolizumab plus bevacizumab, sintilimab plus a bevacizumab biosimilar, lenvatinib, and sorafenib (15–17). For this specific patient, the regimen of camrelizumab combined with apatinib was selected after considering factors such as pharmacoeconomics and drug accessibility. Compared to sorafenib, the combination of camrelizumab and apatinib has demonstrated superior overall survival (OS: 22.1 vs. 15.2 months, P < 0.001) and progression-free survival (PFS: 5.6 vs. 3.7 months, P < 0.001) (18). Current guidelines for the first-line treatment of unresectable hepatocellular carcinoma emphasize immunotherapy as the cornerstone of systemic regimens. While certain international guidelines, such as those issued by the American Society of Clinical Oncology (ASCO) and the European Society for Medical Oncology (ESMO), recommend the combination of atezolizumab and bevacizumab as a first-line option, a meta-analysis has demonstrated that camrelizumab plus apatinib provides comparable survival benefits to the atezolizumab-bevacizumab regimen in the first-line setting for unresectable hepatocellular carcinoma. Therefore, the selection of the camrelizumab-apatinib combination for this patient was both rational and appropriate (19).

As another important local treatment modality, radiotherapy inhibits tumor growth through radiation. The combination of radiotherapy and TACE offers better tumor control compared to either radiotherapy or TACE alone (20).

In a Chinese comparative study of TACE combined with radiotherapy versus TACE alone for unresectable hepatocellular carcinoma, the response rates were 53.7% (43/80) in the TACE group and 71.8% (58/78) in the combination therapy group (p < 0.05). The 1-, 2-, and 3-year survival rates were 58.75%, 36.25%, and 16.25% in the TACE group, compared to 78.48%, 55.12%, and 25.64% in the combination therapy group, respectively (p < 0.05) (21). Therefore, the selection of TACE combined with radiotherapy for this patient has a theoretical basis. During the ongoing maintenance treatment with immunotherapy combined with targeted therapy, the patient’s general condition was acceptable, tumor assessment showed stable disease (SD), and survival has reached 10 months.

However, potential adverse effects such as radiation-induced liver disease must be considered during radiotherapy, necessitating precise target delineation and appropriate radiation doses (22).

**Figure 5:**
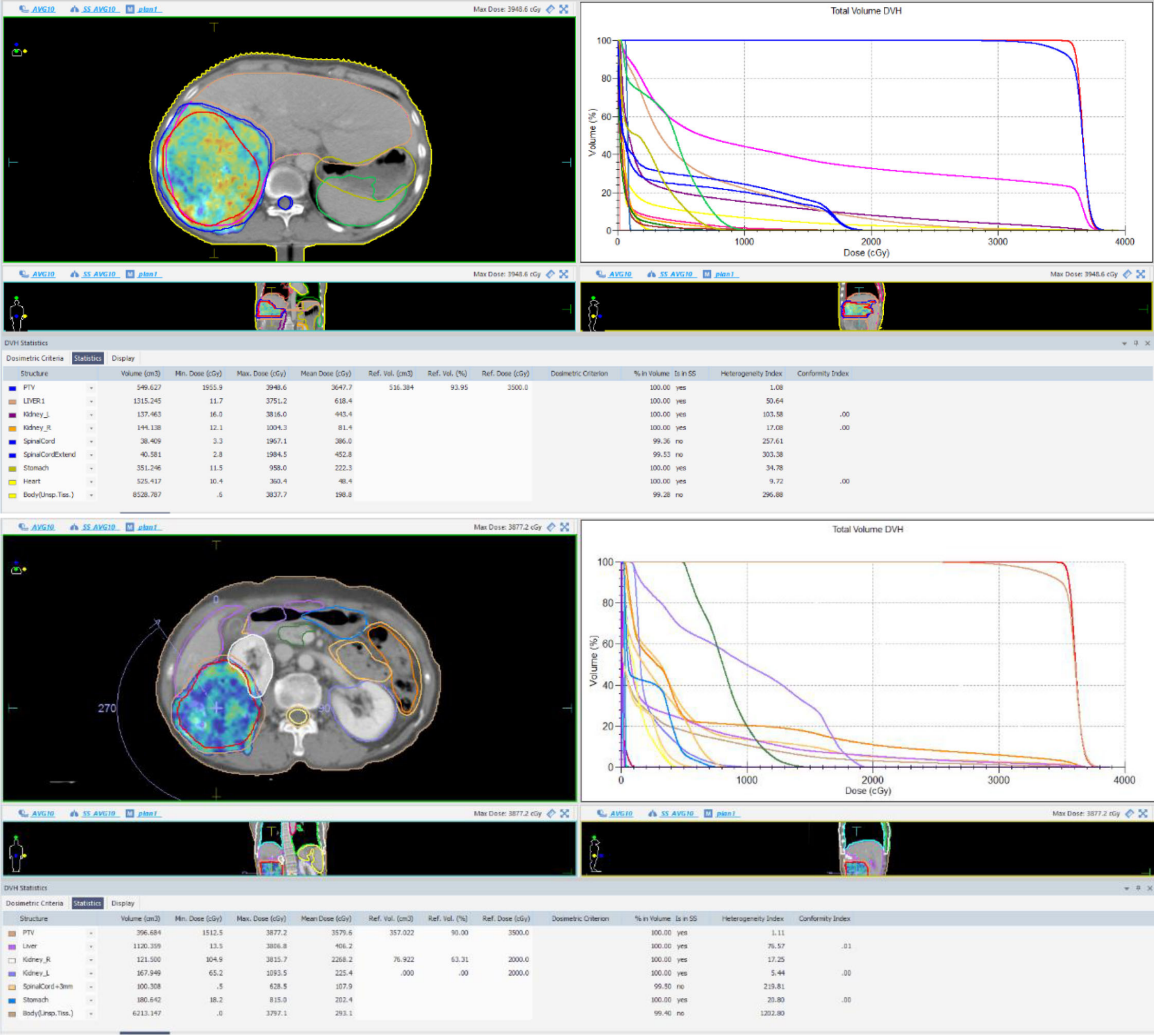
Patient target volume, DVH, and organs at risk doses for the two radiotherapy sessions (top: first session; bottom: second session).

For radiotherapy of hepatocellular carcinoma lesions, radiation-induced liver disease (RILD) is a critical concern. RILD typically occurs within 2 weeks to 4 months after conventional fractionation radiotherapy with a total liver dose of 30–35 Gy (23). It often manifests as anicteric hepatomegaly, ascites, and elevated liver enzymes, particularly alkaline phosphatase. To minimize the risk of RILD, a comprehensive pre-radiotherapy assessment of the patient’s liver function was conducted to rule out contraindications to radiotherapy (the patient had Child-Pugh class A liver function and no viral hepatitis), and an individualized radiotherapy plan was developed. Due to the large volume of the hepatocellular carcinoma lesion, whole-lesion radiotherapy posed a risk of acute liver injury; therefore, the radiotherapy team ultimately decided to deliver the treatment in two separate parts. Studies have shown that when the total liver radiotherapy dose exceeds 35 Gy, the risk of RILD is 44% (24). The total dose per course was limited to 35 Gy, while ensuring a residual liver volume greater than 700 ml to reduce the likelihood of RILD. Given the two-part radiotherapy approach, the cumulative dose to adjacent normal organs increased. Therefore, strict dose constraints for organs at risk (OARs) were established to ensure that the total dose received by these organs did not exceed the planned upper limits (details of normal tissue doses during radiotherapy are shown in **Figure 5**). Throughout the radiotherapy course, the patient’s liver function was regularly monitored to determine whether adjustments to the treatment plan or pharmacological interventions were necessary.

Renal invasion by hepatocellular carcinoma (HCC) represents an exceptionally rare occurrence in clinical oncology, with only six comparable cases identified in the literature to date (25–30).Compared to the previously reported cases, the present case demonstrates several distinctive features:

1. The patient presented in relatively good general condition at initial diagnosis, with no specific chief complaints or overt clinical manifestations. In contrast, some reported cases described symptoms such as back pain or hematuria upon first presentation (25–27, 29).
2. At initial diagnosis, the disease was confined to HCC with direct invasion of the right kidney, without evidence of distant metastasis. Several existing reports indicate that patients often present with metastases to distant organs such as the brain or lungs at the time of diagnosis (26, 28).
3. Since the initiation of treatment, the patient has survived for over four months with disease, exceeding the survival duration reported in most comparable cases (25–28, 30). Previous reports suggest that survival after initial treatment is frequently less than three months, indirectly indicating a poor prognosis associated with renal invasion by HCC (25–27). According to subsequent follow-up, as of January 2026, the patient has survived for more than 10 months since the first visit to our hospital. The size of the liver lesion remained similar to previous, with an overall assessment of stable disease (SD). Meanwhile, the patient’s liver function was Child-Pugh class A, and blood pressure was maintained within the normal range.
4. Under a comprehensive treatment strategy, the patient has not only achieved survival benefit but has also shown a marked downward trend in serum AFP levels during therapy, along with significant tumor shrinkage on imaging, indicating effective disease control. Furthermore, this case highlights a tailored, integrated therapeutic approach that aligns with the characteristics of HCC management in China, offering valuable insights and a potential treatment framework for similar cases in the future.

For this patient, several points require attention:

1. The large primary HCC lesion with direct invasion of the right kidney indicates an advanced stage. Despite current comprehensive treatment, the likelihood of disease progression or new distant metastasis remains high.
2. Due to right renal invasion, there is a potential risk of long-term renal function decline.
3. The possibility of persistent liver function abnormalities following comprehensive treatment including radiotherapy and pharmacotherapy.
4. Management of targeted therapy-related adverse effects (e.g., hypertension) and potential adjustments to the anti-tumor regimen.
5. If disease progression occurs, the efficacy and safety of adding a targeted agent with a different mechanism to the existing regimen, forming a triple-drug anti-tumor therapy, warrant evaluation.

## Conclusion

This case report presents an exceptionally rare instance of hepatocellular carcinoma with direct renal invasion, managed through a tailored multimodal strategy combining locoregional and systemic therapies. The observed tumor regression and significant decline in AFP levels underscore the potential efficacy of an integrated approach incorporating TACE, fractionated radiotherapy, and combination immunotherapy (camrelizumab plus apatinib) for advanced HCC with uncommon extrahepatic involvement. This experience highlights the importance of individualized, comprehensive treatment plans in such complex presentations and suggests that aggressive multimodal management may confer meaningful disease control and survival benefit, warranting further consideration in similar clinical scenarios.

## Data Availability

The original contributions presented in the study are included in the article/Supplementary Material. Further inquiries can be directed to the corresponding author.

## References

[1] Zhou M Wang H Zeng X Yin P Zhu J Chen W . Mortality, morbidity, and risk factors in China and its provinces, 1990-2017: a systematic analysis for the Global Burden of Disease Study 2017. Lancet. (2019) 394:1145–58. doi: 10.1016/s0140-6736(19)30427-1. PMID: 31248666 PMC6891889

[2] Terada T Maruo H . Unusual extrahepatic metastatic sites from hepatocellular carcinoma. Int J Clin Exp Path. (2013) 6:816. 23638212 PMC3638091

[3] Hyun YS Choi HS Bae JH Jun DW Lee HL Lee OY . Chest wall metastasis from unknown primary site of hepatocellular carcinoma. World J Gastroenterol. (2006) 12:2139. doi: 10.3748/wjg.v12.i13.2139. PMID: 16610073 PMC4087701

[4] Xie D Shi J Zhou J Fan J Gao Q . Clinical practice guidelines and real-life practice in hepatocellular carcinoma: a Chinese perspective. Clin Mol Hepatol. (2023) 29:206–16. doi: 10.3350/cmh.2022.0402. PMID: 36545708 PMC10121293

[5] de Martel C Maucort-Boulch D Plummer M Franceschi S . World-wide relative contribution of hepatitis B and C viruses in hepatocellular carcinoma. Hepatology. (2015) 62:1190–200. doi: 10.1007/978-3-319-54567-7_16. PMID: 26146815 PMC5019261

[6] Hanif H Ali MJ Susheela AT Khan IW Luna-Cuadros MA Khan MM . Update on the applications and limitations of alpha-fetoprotein for hepatocellular carcinoma. World J Gastroenterol. (2022) 28:216–29. doi: 10.3748/wjg.v28.i2.216. PMID: 35110946 PMC8776528

[7] Kim DY Han KH . Epidemiology and surveillance of hepatocellular carcinoma. Liver Cancer. (2012) 1:2–14. doi: 10.1159/000339016. PMID: 24159567 PMC3747543

[8] Feng H Li B Li Z Wei Q Ren L . PIVKA-II serves as a potential biomarker that complements AFP for the diagnosis of hepatocellular carcinoma. BMC Cancer. (2021) 21:401. doi: 10.1186/s12885-021-08138-3. PMID: 33849479 PMC8045263

[9] Ngan H Peh WCG . Arteriovenous shunting in hepatocellular carcinoma: its prevalence and clinical significance. Clin Radiol. (1997) 52:36–40. doi: 10.1016/s0009-9260(97)80303-0. PMID: 9022578

[10] Zheng J Wang S Xia L Sun Z Chan KM Bernards R . Hepatocellular carcinoma: signaling pathways and therapeutic advances. Signal Transduct Target Ther. (2025) 10:35. doi: 10.1038/s41392-024-02075-w. PMID: 39915447 PMC11802921

[11] Chen C Wang Z Ding Y Qin Y . Tumor microenvironment-mediated immune evasion in hepatocellular carcinoma. Front Immunol. (2023) 14:1133308. doi: 10.3389/fimmu.2023.1133308. PMID: 36845131 PMC9950271

[12] Reig M Forner A Rimola J Ferrer-Fàbrega J Burrel M Garcia-Criado Á . BCLC strategy for prognosis prediction and treatment recommendation: The 2022 update. J Hepatol. (2022) 76:681–93. doi: 10.1016/j.jhep.2021.11.018. PMID: 34801630 PMC8866082

[13] Liang B Makamure J Shu S Zhang L Sun T Zheng C . Treatment response, survival, and safety of transarterial chemoembolization with calliSpheres((R)) microspheres versus conventional transarterial chemoembolization in hepatocellular carcinoma: A meta-analysis. Front Oncol. (2021) 11:576232. doi: 10.3389/fonc.2021.576232. PMID: 33796448 PMC8008112

[14] Quartuccio N Ialuna S Scalisi D D'Amato F Barcellona MR Bavetta MG . The influence of additional treatments on the survival of patients undergoing transarterial radioembolization (TARE). Curr Oncol. (2024) 31:1504–14. doi: 10.3390/curroncol31030114. PMID: 38534947 PMC10969045

[15] Finn RS Qin S Ikeda M Galle PR Ducreux M Kim TY . Atezolizumab plus bevacizumab in unresectable hepatocellular carcinoma. N Engl J Med. (2020) 382:1894–905. doi: 10.1056/nejmoa1915745. PMID: 32402160

[16] Ren Z Xu J Bai Y Xu A Cang S Du C . Sintilimab plus a bevacizumab biosimilar (IBI305) versus sorafenib in unresectable hepatocellular carcinoma (ORIENT-32): a randomised, open-label, phase 2–3 study. Lancet Oncol. (2021) 22:977–90. doi: 10.1016/s1470-2045(21)00252-7. PMID: 34143971

[17] Qin S Bi F Gu S Bai Y Chen Z Wang Z . Donafenib versus sorafenib in first-line treatment of unresectable or metastatic hepatocellular carcinoma: a randomized, open-label, parallel-controlled phase II-III trial. J Clin Oncol. (2021) 39:3002–11. doi: 10.1200/jco.21.00163. PMID: 34185551 PMC8445562

[18] Qin S Ren Z Meng Z Chen Z Chai X Xiong J . Camrelizumab in patients with previously treated advanced hepatocellular carcinoma: a multicentre, open-label, parallel-group, randomised, phase 2 trial. Lancet Oncol. (2020) 21:571–80. doi: 10.1016/s1470-2045(20)30011-5. PMID: 32112738

[19] Chen JJ Jin ZC Luo B Wang YQ Li R Zhu HD . New first-line immunotherapy-based therapies for unresectable hepatocellular carcinoma: A living network meta-analysis. J Clin Transl Hepatol. (2024) 12:15–24. doi: 10.14218/jcth.2023.00188. PMID: 38250466 PMC10794275

[20] Seong J . Challenge and hope in radiotherapy of hepatocellular carcinoma. Yonsei Med J. (2009) 50:601–12. doi: 10.3349/ymj.2009.50.5.601. PMID: 19881961 PMC2768232

[21] Chen WJ Yuan SF Zhu LJ Sun XN Zheng W . Three-dimensional conformal radiotherapy in combination with transcatheter arterial chemoembolization in the treatment of hepatocellular carcinoma. J Buon. (2014) 19:692–7. doi: 10.1007/bf00647234. PMID: 25261654

[22] Ohri N Dawson LA Krishnan S Seong J Cheng JC Sarin SK . Radiotherapy for hepatocellular carcinoma: new indications and directions for future study. J Natl Cancer Inst. (2016) 108. doi: 10.1093/jnci/djw133. PMID: 27377923 PMC6279296

[23] Koay EJ Owen D Das P . Radiation-induced liver disease and modern radiotherapy. Semin Radiat Oncol. (2018) 28:321–31. doi: 10.1016/j.semradonc.2018.06.007. PMID: 30309642 PMC6402843

[24] Dawson LA Ten Haken RK . Partial volume tolerance of the liver to radiation. Semin Radiat Oncol. (2005) 15:279–83. doi: 10.1016/j.semradonc.2005.04.005. PMID: 16183482

[25] Fukushima M Isoyama E Sakaridani N Sanematsu H Kadowaki H Hirakawa S . Renal metastasis originating from liver cancer. Nihon Hinyokika Gakkai Zasshi Japanese J Urol. (1996) 87:710–3. doi: 10.5980/jpnjurol1989.87.710. PMID: 8709449

[26] Mezawa S Homma H Doi T Takada K Kukitsu T Kinebuchi M . Re: spontaneous rupture of renal metastasis of hepatocellular carcinoma: management by emergency transcatheter arterial embolization. Cardiovasc Interventional Radiol. (2001) 24:143–4. doi: 10.1007/s002700000381. PMID: 11446334

[27] Aron M Nair M Hemal A . Renal metastasis from primary hepatocellular carcinoma: a case report and review of the literature. Urol Int. (2004) 73:89–91. doi: 10.1159/000078812. PMID: 15263801

[28] Ong KWK Joseph B Gyomber DV Bolton DM Lawrentschuk N . Nephrectomy for a renal metastasis of undiagnosed hepatocellular carcinoma arising from an orthotopic liver transplant undertaken for cryptogenic cirrhosis. Korean J Urol. (2013) 54:715. doi: 10.4111/kju.2013.54.10.715. PMID: 24175048 PMC3806998

[29] Kinoshita O Ichijo Y Yoneda M Ikai A Yamashita T . Spontaneous rupture of renal metastasis from hepatocellular carcinoma. Case Rep Surg. (2017) 2017:8607061. doi: 10.1155/2017/8607061. PMID: 28611931 PMC5458372

[30] Yamanaka R Sekino Y Babasaki T Kitano H Ikeda K Goto K . Renal metastasis from primary hepatocellular carcinoma: a case report. Int Cancer Conf J. (2020) 9:141–5. doi: 10.1007/s13691-020-00409-3. PMID: 32582519 PMC7297933

